# Editorial: Precision Medicine in Pulmonary Diseases—Same Principles, New Approach

**DOI:** 10.3389/fmed.2021.821013

**Published:** 2021-12-20

**Authors:** Paula Tejera, Jose Jardim, Maria E. Laucho-Contreras

**Affiliations:** ^1^Department of Environmental Health, Harvard T.H. Chan School of Public Health, Harvard University, Boston, MA, United States; ^2^Escola Paulista de Medicina, São Paulo, Brazil; ^3^Fundación Neumológica Colombiana, Bogotá, Colombia

**Keywords:** precision medicine, COPD - chronic obstructive pulmonary disease, NSCLC - lung adenocarcinoma - EGFR - ALK - BRAF - KRAS - RET - MET - PD-L1 - ROS1, big data & analytics, wood smoke exposition, lung function

Precision medicine is a health care approach that considers differences in genetic, biomarkers, environmental or even lifestyle factors, to classify patients with similar clinical characteristics, into subpopulations or *endotypes* that differ in disease mechanisms and response to treatment. Based on that particular endotype's characteristics, precision medicine can offer a far more customized and specific treatment strategy and prevention, improving clinical outcomes and patient safety (1, 2).

The development of precision medicine for the management of pulmonary diseases such as asthma, chronic obstructive pulmonary disease, idiopathic pulmonary fibrosis, etc., has been mainly limited by our ability to capture heterogeneity and define unique and meaningful endotypes (3, 4). In this Research Topic we focus on the importance of identifying endotypes in the pulmonary medicine, with the final objective of highlighting new therapeutic approaches, potential prognoses, and diagnoses, genetic or therapeutic biomarkers, and/or new pathophysiological mechanisms in respiratory diseases.

Ramirez-Venegas et al.; try to answer the question of the differences in the development and evolution of COPD in non-smokers (secondary to exposure to wood smoke). The authors in this review show a lower rate of decline in FEV_1_ in patients exposed to wood smoke compared to those exposed to cigarette smoke (23 vs. 42 ml, respectively, *P* <0.001) in a Mexican cohort, an effect that the authors named as the “Slow Horse Racing Effect.” Ramirez-Vanegas et al., also discuss possible explanations that consider the size of particles and the anatomical location of tissue damage, rather than different inflammatory mechanisms, as has been proposed in previous publications (5). This approach is interesting, but it must be considered within the limitations associated to this review (data mainly from articles of their own group), and the differences between the studies that were evaluated to make the final manuscript. The study by Ramirez-Vanegas et al. moves us away from the approach “one size for all,” shedding light on the importance of the heterogeneity of the diseases and bringing us closer to the implementation of precision medicine in obstructive diseases. A new taxonomy of pulmonary diseases based on causative underlying molecular mechanisms, may help to rise to the challenge of precision medicine in the framework of respiratory diseases, by tailoring treatments that meet the patient's medical needs.

The availability of vast amounts of multi-omics data generated from a large set of samples represent an unique opportunity for the transformation of medicine into precision medicine (6). However, as it is rightly stated in Raita et al., “big data” alone are useless. In their work, the authors discussed the instrumental role that data science plays in enabling the transition toward precision medicine, by integrating machine learning algorithms, that handle quantitatively “big data,” with causal reasoning and knowledge (through causal inference). By means of this integration, data science will enable clinicians to understand how complex systems work, and to address the fundamental causal questions in precision medicine that will assist them in making informed decisions, and lastly improve health outcomes of their patients. Limitations for the implementation of data science approaches in clinical practice are also discussed in the review. The authors also provide useful educational resources for those clinicians interested in learning the fundamentals of data science.

Emphysema and pulmonary fibrosis can coexist, resulting in clinical syndrome known as combined pulmonary fibrosis and emphysema (CPFE), characterized by dyspnea, upper-lobe emphysema, lower-lobe fibrosis, abnormalities of gas exchange, and worse clinical outcomes. The etiology and pathophysiology of CPFE remain unclear and the question of whether CPFE is the result of the interaction between COPD and IPF, or on the contrary it is and independent entity itself remains open. In their study, Guzmán-Vargas et al., try to answer this question by investigating the genetic profile associated with the risk of the individual and combined diseases. To do so, the authors study the association between genetic variants (SNPs), previously associated with COPD and IPF (FAM13A), rs2736100 (TERT), rs2076295 (DSP), 128 rs5743890 and rs111521887 (TOLLIP), and the risk of CPFE in a mestizo Mexican population. The authors pointed out a differential genomic profile between COPD patients with emphysema, IPF, and CPFE that could represent different underlying mechanisms involved in the pathogenesis of the three diseases. Nevertheless, small sample size is an important limitation of this study and its conclusions must be interpreted with caution.

Finally, it is worth highlighting the work by Gu et al., where the authors demonstrated the correlation between the expressed levels of Ki-67 and the epidermal growth factor receptor (EGFR)- or- Kristen rat sarcoma viral oncogene homolog (KRAS) mutant state, in patients with non-squamous cell lung cancer (NSCLC) receiving first-line treatment. In this interesting study, the usefulness of the use of nomograms with multiple covariates is shown to predict response to treatment and overall survival. However, it is necessary to consider the limitations of a retrospective, single-center study, without an external validation cohort.

In general lines, after reviewing this very interesting topic, we conclude that achieving precision medicine in pulmonary diseases will be determined by new approaches, based on our ability to look for the differences, for the necessary pieces in the construction of customized solutions that will benefit patients always following an old and important principle in medical care: *Primum Non Nocere*.

## Author Contributions

All authors listed have made a substantial, direct, and intellectual contribution to the work and approved it for publication.

## Conflict of Interest

ML-C and JJ currently work at GSK. The remaining author declares that the research was conducted in the absence of any commercial or financial relationships that could be construed as a potential conflict of interest.

## Publisher's Note

All claims expressed in this article are solely those of the authors and do not necessarily represent those of their affiliated organizations, or those of the publisher, the editors and the reviewers. Any product that may be evaluated in this article, or claim that may be made by its manufacturer, is not guaranteed or endorsed by the publisher.
